# Familial Mediterranean fever, from pathogenesis to treatment: a contemporary review

**DOI:** 10.3906/sag-2008-11

**Published:** 2020-11-03

**Authors:** Abdurrahman TUFAN, Helen J LACHMANN

**Affiliations:** 1 Department of Internal Medicine, Division of Rheumatology, Gazi University, Ankara Turkey; 2 National Amyloidosis Centre, Royal Free London NHS Foundation Trust and University College London, London UK

**Keywords:** Familial Mediterranean fever, genetics, pathogenesis, diagnosis, clinical features, treatment, prognosis

## Abstract

Familial Mediterranean fever (FMF) (OMIM #249100) is the most common hereditary autoinflammatory disease in the world. FMF is caused by gain of function mutations of MEFV gene which encodes an immune regulatory protein, pyrin. Over the last few years, we have witnessed several new developments in the pathogenesis, genetic testing, diagnosis, comorbidities, disease related damage and treatment approaches to FMF. Elucidation of some of the pathogenic mechanisms has led to the discovery of pathways involved in inflammatory, metabolic, cardiovascular and degenerative diseases. The use of next generation sequencing in FMF has revealed many new gene variants whose clinical significance may be clarified by developing functional assays and biomarkers. Clinically, although FMF is considered an episodic disease characterized by brief attacks, recent systematic studies have defined several associated chronic inflammatory conditions. Colchicine is the mainstay of FMF treatment, and interleukin (IL)-1 antagonists are the treatment of choice in refractory or intolerant cases. Experience of IL-1 antagonists, anakinra and canakinumab, is now available in thousands of colchicine resistant or intolerant FMF patients. In this contemporary review, we surveyed current FMF knowledge in the light of these recent advances.

## 1. Introduction 

Familial Mediterranean fever (FMF) is the most common hereditary autoinflammatory disease in the world. It primarily affects populations originated from East Mediterranean territory though patients are reported worldwide. FMF is caused by gain of function mutations in the MEFV gene, which encodes a protein called pyrin which has regulatory functions on the innate immune system. For many years the high carrier frequency for MEFV mutations in Middle Eastern populations was thought to confer a selective advantage for an unknown human threat and recent data suggests that it includes the cause of the “black death”, *Yersinia pestis* [1]. 

FMF is characterized mainly by recurrent short-lived episodes of peritonitis, pleuritis, arthritis, rash, and usually with accompanying fever. Recent systematic studies have defined a variety associated inflammatory conditions in the setting of FMF. As genetic testing has become cheaper and widely available it is increasingly used in daily clinical practice. Next generation sequencing (NGS) has identified many new variants but their clinical associations are largely unknown, hampering their interpretation. Functional assays and biomarkers that may aid in diagnosis, assessment of disease severity and treatment are under development [2]. Practical indices quantifying FMF disease severity, activity and recently damage accrual have been developed and validated for clinical use and research purposes [3–5].

Colchicine is the mainstay of FMF treatment, interleukin (IL)-1 antagonists are the treatment of choice in refractory or intolerant cases and new treatment options are under development. The use of IL-1 antagonists, anakinra and canakinumab, has now reached thousands of colchicine resistant or intolerant FMF patients and data on their efficacy and safety are becoming more robust. However, information on their long-term utility in terms of preventing damage and practice guidelines for the rational use are still lacking. 

It is noteworthy that elucidation of some of the mechanisms underlying FMF pathogenesis has led to the discovery of critical immune pathways including intracellular danger sensors, inflammasomes, pyroptosis and NETosis, thereby transforming the understanding of mechanisms and treatment of prevalent inflammatory, metabolic, cardiovascular and degenerative diseases. Studies investigating efficacy of colchicine and IL-1 antagonists on these diseases are promising and expanding [6]. Over the last few years, we have witnessed several new developments in FMF pathogenesis, genetic testing, comorbidities and their management, disease related damage and treatment approaches. In this contemporary review, we presented the current knowledge on FMF in the light of recent advances.

## 2. Literature search strategy

We conducted a literature search on FMF from 1967 to May 2020 using the Medline, Embase, and Cochrane databases and the following terms: ‘pathogenesis’, ‘genetics’, ‘manifestation’, ‘chronic inflammation’, ‘treatment’, ‘complication’, ‘amyloidosis’, ‘prognosis’, and ‘familial Mediterranean fever’. We reviewed abstract archives of the International Society of Systemic Auto-Inflammatory Diseases (ISSAID), European League Against Rheumatism (EULAR), and American College of Rheumatology (ACR) congresses for unpublished data on specific topics of interests such as colchicine resistance and intolerance. All the articles we surveyed were included in Endnote library, and duplicate articles were removed from the library. We limited our search to articles published in the English language.

## 3. Genetics and pathogenesis

Unusually for a disease largely inherited in an autosomal recessive fashion, FMF results from gain of function mutations of Mediterranean fever gene (MEFV), located on chromosome 16 (16p13.3) [7]. MEFV encodes for a 781 amino acid protein, pyrin (‘pyrin’ or ‘marenostrin’, TRIM20) which exist in several isoforms both in cytoplasm and nucleus [8]. The exact function of pyrin in nucleus is largely unknown. Pyrin has at least four functional domains; PYD, bBOX, CC and B30.2/SPRY. Upon activation, pyrin oligomerizes with other cellular proteins, forming a macromolecular complex called ‘pyrin inflammasome’ that activates caspase-1 which, in turn, mediates the release of pro-inflammatory IL-1β and IL-18 from their inactive precursors and pyroptosis via the gasdermin D pathway [1, 9]. 

Cytoplasmic pyrin interacts with microtubules in cell skeleton and is a member of cytosolic pattern recognition receptors (PRRs) which are responsible for the initiation of rapid innate immune responses by sensing endogenous danger- or exogenous pathogen- associated molecular patterns (DAMPs and PAMPs). However, unlike other receptors, pyrin does not recognize DAMPs/PAMPs directly, but rather detects alterations in cytoplasmic homeostasis, incited by harmful stimuli, termed “homeostasis-altering molecular processes” (HAMPs). These modify RhoA GTPase within cells [10]. In physiologic conditions, RhoA GTPase activates the serine-threonine kinases; PKN1 and PKN2, that bind and phosphorylate pyrin. Phosphorylated pyrin binds to inhibitory 14-3-3 proteins and this process retains pyrin in an inactive state that prevents formation of the pyrin inflammasome. In FMF, mutations in MEFV gene impair interaction of pyrin with microtubules, PKN and 14-3-3 proteins facilitating formation of a proinflammatory pyrin inflammasome. When the pyrin inflammasome assembled, it activates caspase-1 to process pro-IL-1β and pro-IL-18 to their mature forms IL-1β and IL-18, respectively, and cells undergo an inflammatory death termed pyroptosis. Over activation of the pyrin inflammasome, and the resulting inflammation drives typical febrile inflammatory attacks observed in FMF [10,11]. 

IL-1β stimulates expression of genes involved in the entire IL-1 pathway, thereby enhancing its own production, contributing to an inflammatory burst [12]. Expression of pyrin can be upregulated by various cytokines: interferon (IFN)-γ, Tumor Necrosis Factor (TNF)-α, IL-4, and IL-10 as well as Lipopolysaccharides (LPS) [10]. Pyrin is mainly expressed in innate cells – including granulocytes, cytokine-activated monocytes, dendritic cells, and synovial as well as serosal fibroblasts – which explains the typical organ sites involved in FMF The Human Protein Atlas (2020). MEFV [online]. Website u299b [accessed 00 Month Year].. Although episodic nature of FMF is not completely understood, during attacks neutrophil extracellular traps (NETs), i.e. chromatin ﬁlaments ‘decorated’ with neutrophilic proteins and captured IL-1β, are formed, restricting further generation of IL-1β by a negative feedback mechanism, which may explain the self-limited nature of FMF attacks [13].

MEFV gene has 10 exons and there are more than 370 variants identified to date. The number of variants are increasing with use of genome sequencing Infevers (2020). MEFV sequence variants [online]. Website u299f [accessed 00 Month Year].. Most of the pathogenic/likely pathogenic variants are located on exon 10 which encodes B30.2/SPRY domain, responsible for the activation of caspase-1. The most common MEFV variant is M694V (c.2080A>G) in FMF endemic areas. Other frequent exon 10 variants are M694I (c.2082G>A), V726A (c.2177T>C) and M680I (c.2040G>C and c.2040G>A) and these variants constitute almost 75% of all FMF patients (Table 1) [14,15]. Carriage of these variants is associated with the typical clinical phenotype of FMF and with more severe disease [16]. Benign and likely benign variants are usually located on exon 2 and generally do not cause typical phenotype of FMF. Up to two thirds of registered variants distributed over the entire gene are either not classified or classified as variant of uncertain significance (VUS) due to their unknown clinical association [17]. 

**Table 1:** Examples of commonly found MEFV gene variants with respect to their clinical association.

Benign	Likely benign	VUS	Likely pathogenic	Pathogenic
D102D	R75Q	E148Q	S208T	M680IG>C
G138G	P115T	P369S	F479L	M680I G>A
R202Q	G304R	H478Y	M680L	M694V
R314R	A317T	G678E	I692DEL	M694I
E474E	A457V	T681I	M694L	V726A
D510D	I591M	I720M	K695R	
P588P	V690L	V722M	K695N	
S675N	I772V	A744S	R761H	

Infevers (2020). MEFV sequence variants [online]. Website https://infevers.umai-montpellier.fr/web/search.php?n=1 [accessed 30 May 2020].

FMF is inherited as autosomal recessive pattern, however approximately 30% of patients harbour single pathogenic variant (monoallelic disease) [18]. In FMF prevalent countries and high rate of consanguineous marriages accumulation of individuals from consecutive generations might be perceived as dominant inheritance which is indeed pseudo-dominant transmission. However, some of MEFV variants with true dominant inheritance have also been defined [19,20]. Symptom-free individuals with one identifiable pathogenic MEFV gene variant are called ‘asymptomatic carriers’ of the disease, and symptom-free individuals harbouring two pathogenic variants are defined as ‘phenotype 3’ [21]. 

## 4. Epidemiology

FMF is prevalent in populations originating from the Eastern Mediterranean region – namely, Turks, Jews, Arabs, and Armenians. However hundreds of patients are reported from Europe, North America and Japan [22,23], and this figure is expected to increase due to massive population movements from endemic areas to these latter regions over the last two decades. FMF’s prevalence varies from 1:500 to 1:1,000 in endemic countries [22] with the highest reported prevalence of 1/395, from central Anatolia region [24]. Considering disease prevalence and population size, Turkey has the highest number of FMF patients in the world followed by Israel and Armenia [22]. 

## 5. Clinical picture

FMF is characterized by recurrent short-lived inflammatory attacks which resolve spontaneously within 1–3 days. The well-known, canonical manifestations of FMF are fever, serositis, arthritis and erysipelas like erythema [25]. Additionally, a wide array of clinical presentations – such as neurologic, thrombotic, and ocular disease – have been reported in FMF settings and referred to as ‘noncanonical manifestations’ [26]. Some FMF patients may develop complications of disease in their follow up and some others may initially present with them, typical example of the latter is the presentation with amyloid A (AA) amyloidosis in the absence of classical symptoms which is termed as phenotype 2. 

FMF attacks usually start from early childhood and 80%–90% of patients become symptomatic before the age of 20 years [25]. Initial manifestations of the disease after age 40 are fairly rare, and often characterized by a mild disease course [27]. FMF patients generally recognize provoking trigger – such as emotional stress, cold exposure, menstruation, or travel – before the onset of symptoms [28,29]. Early or premonitory symptoms such as loss of appetite, irritability and extremity numbness are described by almost 50% of patients hours before the onset of full blown attacks [30]. These prodromal symptoms are more common in men and before the onset of peritonitis and arthritis attacks [31]. Many individuals describe a typical individual recurrent attack pattern, which may help to differentiate attacks from other conditions, such as acute appendicitis or cholecystitis. 

Frequency of type of FMF manifestations differ between studies according to the enrolled age group, geographic region and ethnic population. However, fever and peritonitis are by far the most common manifestations, reported in more than 90% of patients in all age and ethnic groups [16,22]. FMF associated fever usually starts suddenly, spikes within hours and resolves spontaneously on the same day. The pattern of fever may help to differentiate FMF from infectious causes and other autoinflammatory diseases [32]. Fever may occur alone or accompany other FMF manifestations. In rare instances, fever might be the only or initial symptom which could be handled as fever of unknown origin [33,34]. 

**Figure 1:**
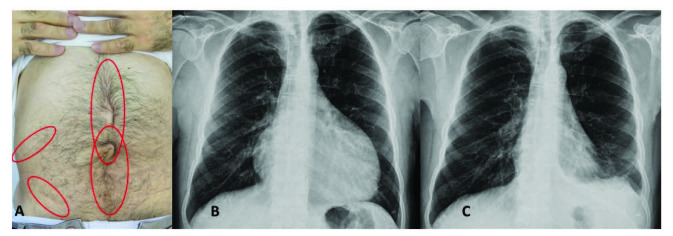
(A) A patient with scars from multiple abdominal surgeries, (B) chest X-ray showing massive pericardial effusion, (C) chest X-ray of the same patient after treatment with anakinra.

Peritoneal attacks start localized and spread rapidly involving the entire abdomen. Peritoneal inflammation causes a typical ileus picture and patients suffer from crippling stomach pain. Physical examination reveals rigidity of the abdominal muscles, rebound tenderness, and loss of bowel sounds – an emergency scenario clinically indistinguishable from the surgical causes of acute abdomen. Thus, a history of unrevealing abdominal surgery is common among FMF patients (Figure 1A). In suspected cases, imaging studies may help to exclude surgical pathologies. A mild rebound diarrhea may follow abdominal attacks. Despite their dramatic presentation, all signs and symptoms of peritonitis completely resolve without a sequela over 24–72 h, though chronic ascites and peritoneal adhesions have been reported rarely [35]. 

Less than 50% of patients experience pleural attacks, which manifest as sudden-onset, unilateral pleuritis and may occur either alone or in combination with peritonitis and fever. Patients describe typical severe pleuritic chest pain and, upon examination, their breath sounds are diminished on the affected side of their chest. Chest X-rays may reveal a small exudate at the costophrenic angle. Attacks usually resolve completely within one to three days [36,37]. 

Pericarditis is a rare condition observed in 1%–2% of patients [38]. Pericarditis gives symptoms as retrosternal chest pain and dyspnea. Cardiac tamponade may occur very infrequently. An electrocardiogram shows an elevated ST segment, chest X-rays may reveal transient enlargement of the cardiac silhouette (Figures 1B and 1C), and echocardiography shows accumulation of pericardial fluid [39]. Rarely, recurrent pericarditis can be the sole manifestation of FMF, which might be confused with idiopathic pericarditis [40,41]. 

**Figure 2:**
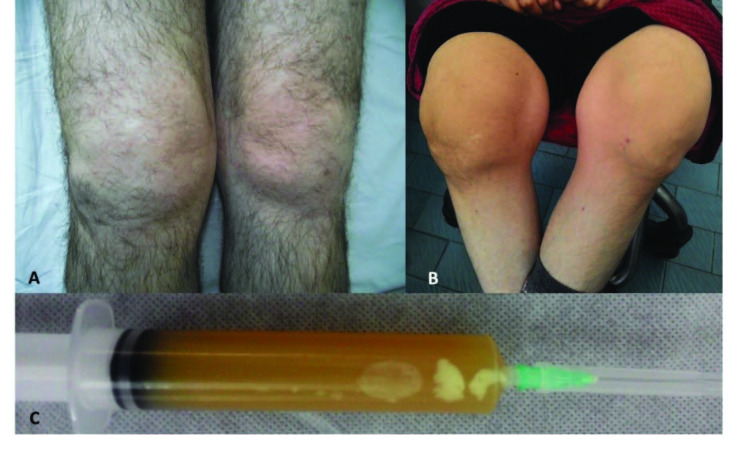
(A) A patient with acute knee arthritis, (B) chronic deforming arthritis involving both knees, (C) joint aspirate showing sterile inflammatory effusion.

**Figure 3:**
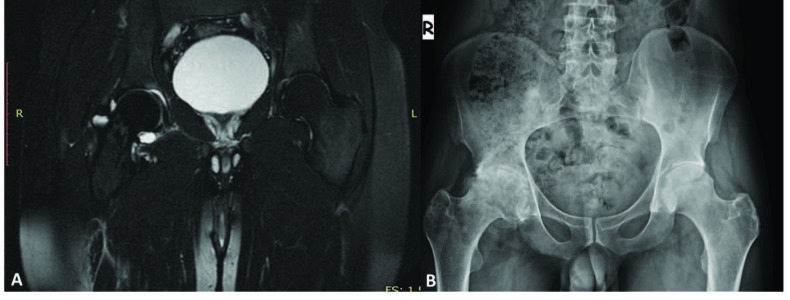
(A) Acute right hip joint arthritis, (B) permanent destruction in the same joint in the long term.

Arthritis is the third most common manifestation of FMF affecting more than 50% of patients. Articular attacks occur as sudden-onset, painful, swollen joints. These attacks involve one or two large joints of the lower extremities and can be accompanied by fever, a condition indistinguishable from septic arthritis (Figures 2A and 2B). Joint aspirate reveals a sterile inflammatory effusion (Figure 2C). Symptoms peak in one to three days and subside spontaneously within a week. Protracted arthritis is observed in fewer than 5% of patients, and permanent damage may occur in a small proportion of patients – particularly when the hip joints are involved (Figure 3). Chronic sacroiliitis is reported in approximately 10% of FMF patients [42]. Articular attacks are more common in patients harbouring an M694V mutation, and it is associated with more severe disease as well as increased risk of AA amyloidosis [43]. 

**Figure 4:**
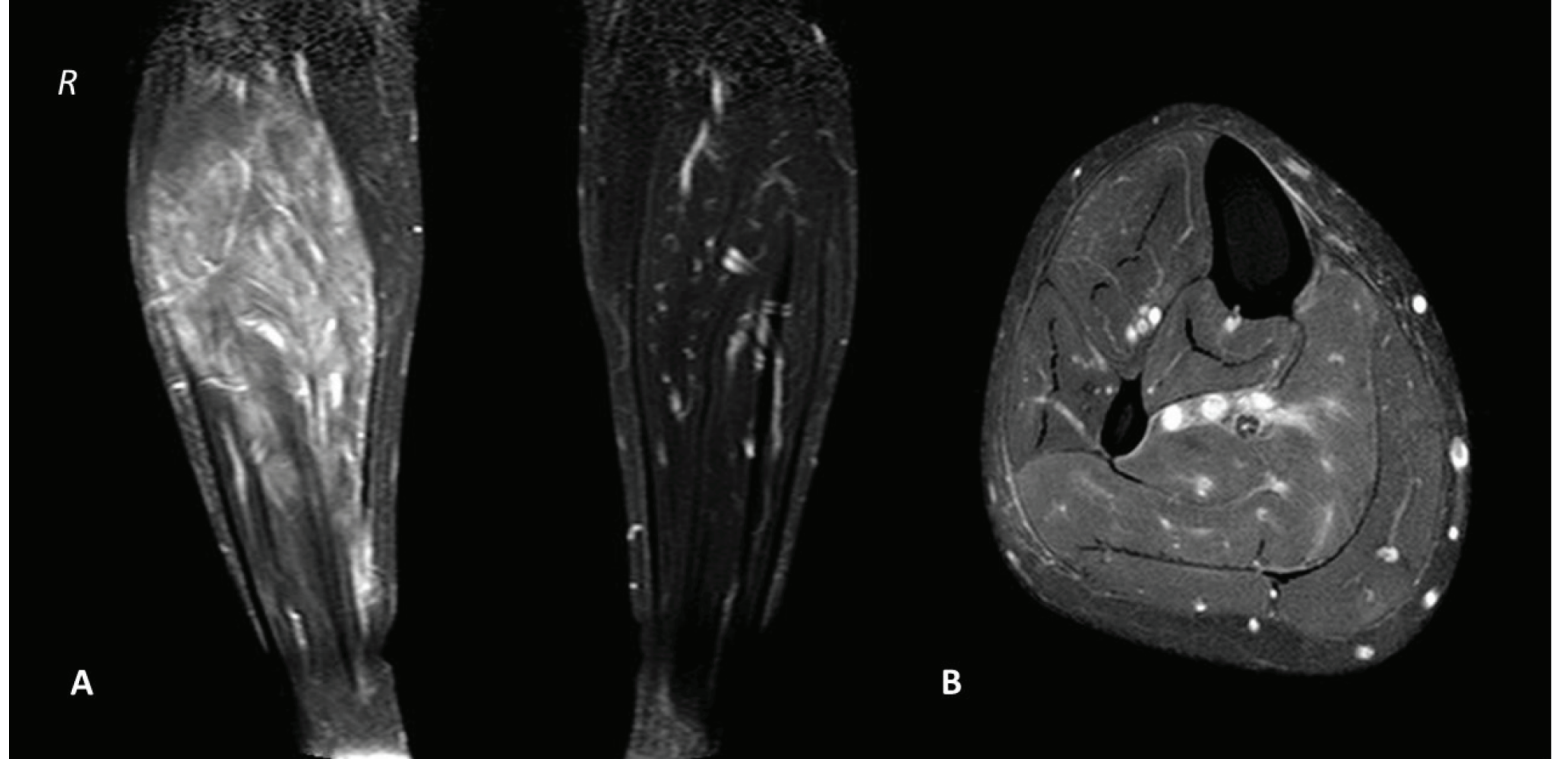
(A) Coronal and (B) axial T2-weighted fat saturation magnetic resonance images of the right calf muscles showing increased intensity and severe swelling due to muscular inflammation.

Muscular complaints are common in FMF patients, particularly those carrying the M694V mutation. The most common muscular symptom is standing myalgia (exertional leg pain) which occurs in calf muscles. Attacks might be evoked by long travel and probably by passive vibration. Myalgia is associated with more severe disease and persistent inflammation [44,45] that can be easily overlooked if not meticulously questioned. The most severe muscular presentation is protracted febrile myalgia (PFM) which is characterized by intense myalgia and fever with prolonged complaints up to 10 weeks. PFM causes a marked acute-phase response, and magnetic resonance imaging reveals muscle oedema (Figure 4). The condition is refractory to colchicine and may even occur despite regular colchicine use [46]. 

**Figure 5:**
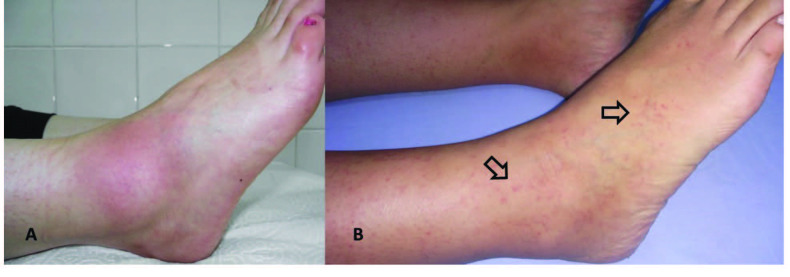
Skin manifestations of familial Mediterranean fever. (A) Erysipelas like erythema (ELE) on the dorsum of the foot, and (B) painful purpura.

The skin manifestations of FMF present as a patchy skin rash resembling typical erysipelas (erysipelas-like erythema, ELE) or as painful purpura resulting from leukocytoclastic vasculitis (Figure 5). Skin attacks usually follow long travel or prolonged walking and occur mostly on the legs, around the ankle, or on the dorsum of the feet. 

A wide array of presentations – including vasculitic, neurologic, thrombotic, ocular, and cochlear disorders– have been reported in FMF patients and defined as ‘non-canonical manifestations’ [26]. However, the prevalence and pathogenetic associations of them with FMF have not been well characterized in most of these disorders, which might also be interpreted as coincidental occurrences [47]. 

It has long been noted that FMF patients and asymptomatic carriers are vulnerable to certain inflammatory diseases, such as spondyloarthritis, Henoch–Schönlein purpura (HSP), and polyarteritis nodosa (PAN) [48]. The increased incidence of these particular diseases in FMF patients can be explained by an enhanced expression of proinflammatory cytokines and microRNAs. Recent publications have expanded this list to include: juvenile idiopathic arthritis, PFAPA, spondyloarthropathies, inflammatory bowel disease, vasculitides (Behçet’s disease, HSP, PAN), glomerulonephritis, skin diseases (psoriasis, hidradenitis suppurativa, contact dermatitis), and demyelinating brain disease [48–52]. Most of these comorbid conditions fall under MHC Class I group diseases (MHC-I-opathies), whereas MHC-II–spectrum (autoimmune) diseases have a comparable prevalence among the general population [48,49]. 

Phenotype 2 FMF is characterized by initial presentation with AA amyloidosis in the absence of other classical manifestations of FMF disease. This phenotype constitutes fewer than 2% of patients in different cohorts [38]. Diagnosis requires genetic analysis and exclusion of other causes of chronic inflammation. 

Although FMF is characterized by intermittent inflammatory attacks, a substantial number of patients have chronic, ongoing inflammation. In a prospective study, only 29% of serum amyloid A (SAA) measurements in FMF patients were less than 3 mg/L, 65% were less than 10 mg/L, and 13% exceeded 50 mg/L [53]. Independent risk factors for chronic inflammation were male sex, M694V homozygosity, musculoskeletal-type attacks, colchicine resistance, and inflammatory comorbidities [54]. Chronic inflammation constitutes a major risk for the development of amyloidosis, kidney failure and other complications of disease [54,55].

## 6. Genetics and environmental factors’ effects on phenotypic variation 

The heterogeneous disease course of FMF results from a complex interplay between genetic and environmental factors. The final disease phenotype primarily determined by the MEFV variants carried [14]. M694V is the most common and the most penetrant variant causing more pronounced caspase-1, IL-1 family cytokine and S100A12 secretion from neutrophils [56]. 

Patients harbouring biallelic exon 10 variants – particularly variants which are homozygous for M694V – exhibit a severe disease phenotype characterized by an earlier age of onset, more frequent attacks, musculoskeletal features, lower colchicine response, chronic inflammation, increased risk of comorbid conditions, and disease complications including infertility, joint damage, and secondary amyloidosis [38, 57–59]. Disease associated with a single mutation (MEFV heterozygotes) typically is milder than in individuals with homozygous or compound heterozygous pathogenic variants, characterized by later onset, less frequent fever/serositis type attacks, more favorable response to colchicine and lower risk of disease complications [60,61]. However this is not always the case and reports exist of more severe single variant disease which might be due to inclusion of high number of patients carrying single heterozygous M694V mutation [62]. Approximately 10%–20% of FMF patients in different cohorts do not carry any identified MEFV mutation though it is contentious whether this condition is FMF-like disease or true FMF with as yet unidentified genetic variations [14]. Nevertheless, after extensive genetic testing there are a number of confirmed mutation negative patients who show a mild disease phenotype similar to those with single heterozygous subjects [63]. 

The symptoms and severity of FMF can vary among affected individuals with the same MEFV variant, even among members of the same family [64] suggesting contributions by a number of modifiers – including other genes, epigenetic and environmental factors. Based on twin studies relative contribution of MEFV, non-MEFV genetic factors and environmental factors on phenotypic variation are estimated at a ratio of 6:1.5:1, respectively [65]. The known MEFV independent genetic modifiers are polymorphisms of IL-1β which is associated with disease severity [66] and harboring alpha/alpha genotype for serum amyloid A protein 1 (SAA1) gene which increases the risk of developing AA amyloidosis without affecting FMF disease characteristics [67]. Although specific alterations have been reported in epigenetics such as microRNAs and DNA methylation, their contribution to phenotypic variation has yet to be clarified [68–70]. The influence of specific environmental factors on FMF phenotype have not been defined, but country of residence is repeatedly reported as an independent modifier with living in Turkey, Armenia and Middle East increasing risk of severe disease and AA amyloidosis [71–74]. 

## 7. Diagnosis

The diagnosis of FMF is made based on the patient history, inflammatory markers and more recently by genetic testing. Several clinical diagnostic criteria sets have been proposed for the diagnosis of FMF. The Tel Hashomer criteria, Livneh criteria and Turkish pediatric criteria all rely on the clinical symptoms, family history and colchicine response. The oldest and maybe the most widely used criteria set is the Tel Hashomer, which includes fever, peritonitis, pleuritis, arthritis, ELE, amyloidosis and family history [25]. In 1997, Livneh et al. created a new set derived from the Tel Hashomer criteria but including exertional leg pain and laboratory findings (inflammatory markers and urinary findings, proteinuria/hematuria) and excluding amyloidosis. Both extended and simplified versions of Livneh criteria have a reported sensitivity and specificity of >95% [75]. In 2009, Turkish paediatric criteria for FMF were developed with the inclusion of family history and clinical characteristics of fever, arthritis, stomach, and chest pain. Children with two or more of these criteria can be diagnosed with FMF with a reported sensitivity and specificity of 86.5% and 93.6%, respectively [76]. 

Clinical criteria are supposed to be sufficient for the diagnosis of FMF in endemic countries [22]. However, diagnosis of FMF can be difficult and delayed with use of clinical criteria, if the physician experience of FMF symptoms is limited and when the patient has hazy or atypical symptoms. After molecular cloning of MEFV, genetic testing become available as a diagnostic adjunct, especially in atypical cases and phenotype 2 patients. Recently, Eurofever/PRINTO group released classification (not diagnostic) criteria combining genetic test results and the following clinical features; attacks lasting 1–3 days, arthritis, abdominal and chest pain [77]. According to Eurofever/PRINTO, individuals with biallelic pathogenic/likely pathogenic variants and one clinical feature are classified as FMF, whereas patients harbouring one pathogenic or likely pathogenic variant or low penetrant biallelic variants are required to have two or more clinical features in order to be classified as having FMF. The performance of three clinical FMF diagnostic criteria and the new Eurofever/PRINTO classification criteria was recently tested in a real-life setting on paediatric patients. All of the criteria sets revealed similar performance in diagnosing or classifying patients with biallelic pathogenic variants, but the Eurofever/PRINTO classification criteria had a somewhat lower sensitivity for patients with monoallelic variants (heterozygotes) [78,79]. 

Genetic testing is recommended in suspicion of FMF in non-endemic countries to support/confirm diagnosis but also give valuable information about the severity of disease, like colchicine response, risk of disease complications and ultimately long-term prognosis [80,81]. Genetic testing can be performed either by Sanger or single gene NGS. ISSAID/European Molecular Genetics Quality Network (EMQN) recommends a limited initial MEFV exon 10 sequencing in patients with suspected of FMF [82]. NGS might reveal many clinically undetermined variants complicating interpretation of test results. A recently reported consensus-driven pathogenicity classification failed to classify almost half of the MEFV variants (42.4%) [83]. Functional assays may ascertain pathogenicity of such variants but these are in their infancy and have not been performed for many of them [81]. Lamkanfi et al. recently developed a functional diagnostic test for FMF, ex vivo colchicine assay, with a sensitivity of 86% and specificity of 100%. The test was based on colchicine’s in vitro inhibitory effect on peripheral blood mononuclear cells for the secretion of IL-1β and IL-18 via stimulation with Clostridium difficile toxin A [2].

Commonly used markers of inflammation leukocyte count, erythrocyte sedimentation rate (ESR), C-reactive protein (CRP), serum amyloid A protein (SAA) and fibrinogen are increased during attacks and should return to their normal values in between attacks. CRP, SAA and fibrinogen are produced from liver upon stimulation with proinflammatory cytokines including IL-1β, IL-6, TNF-α and IFN-γ. SAA is found in trace amounts in plasma (normal range <3 mg/L) and its concentration can rapidly increase up to 1000-fold within 24 h in response to inflammatory stimuli [84]. SAA is the precursor of AA amyloid fibrils and sustained long-term elevation of SAA concentration is the only known prerequisite for the development of AA amyloidosis [53]. Similarly, CRP values can rise dramatically up to hundred-folds after an inflammatory stimulus. In a prospective study, Lachmann et al. showed that both SAA and hs-CRP were massively elevated during all reported clinical attacks of FMF in all patients, with median values of 693 (range of 140–1330) mg/L and 115 (range of 26–296) mg/L, respectively [53]. Hence, SAA is the most sensitive biomarker and is recommended to adjust colchicine dose and minimize the risk of AA amyloidosis. Although acute phase proteins (APRs) are helpful in corroborating clinical attacks and inflammatory activity, they all lack specificity for diagnostic use. S100 proteins are DAMP-family molecules which induce IL-1β release with a positive feedback loop. Massively increased (100-fold) S100A12 serum concentrations during and between attacks are suggested to be very specific for FMF and systemic-onset JIA, which may help diagnose and monitor inflammatory activity [85]. 

Indisputably, the most valuable tool in FMF diagnosis is a proper patient history which should include details of symptoms, past medical history, ethnicity and family history. Currently, MEFV molecular genetic testing is readily available, but test results are not unequivocal, and they need to be evaluated cautiously in the clinical context. If both clinical assessment and the genetic testing are inconclusive and the condition is not better explained by an alternative diagnosis, a therapeutic trial of colchicine is given for 3–6 months to ascertain treatment response. However, it should be noted that the diagnostic sensitivity of this approach is almost 90% but its specificity is as low as 15%, resulting in a high rate of false positive diagnoses [86]. Therefore, under this approach, patients should be monitored for the onset of new symptoms that direct alternative diagnoses while maintaining colchicine. 

## 8. Differential diagnosis of FMF

Several diseases may mimic the symptoms of FMF, and they should be considered during diagnosis and in the case of colchicine nonresponse (Table 2). 

**Table 2:** Differential diagnoses of familial Mediterranean fever.

Mevalonate kinase deficiency (MKD)	Inflammatory bowel disease
TNF receptor-associated periodic syndrome (TRAPS)	Porphyria
Periodic fever, aphthous stomatitis, pharyngitis, and adenopathy (PFAPA)	Median arcuate ligament syndrome
Hereditary angioedema	Intestinal adhesion
Fabry disease	Adrenal failure
Cyclic neutropenia	Chronic recurrent infections
Yao syndrome	Mesothelioma

## 9. Treatment

The goals of treatment in FMF are improving quality of life (QoL), reducing frequency, severity and duration of attacks and prevention of long term damage, particularly AA amyloidosis by minimizing chronic/subclinical inflammation. There is great heterogeneity between individuals for the type and severity of attacks. Hence, treatment should be tailored individually with monitoring attacks and inflammatory markers/proteinuria [87]. FMF treatment can be divided into three parts:

· Attack and AA amyloidosis prophylaxis,

· Treatment of acute episodes,

· Management of comorbid conditions and complications. 

Prior to 1972 and the seminal observation that colchicine prophylaxis decreases FMF attacks and subsequently the risk of AA amyloidosis, more than 50% of patients with FMF died of amyloidosis [25,88]. But even recently AA amyloidosis is a major problem among FMF patients affecting up to 10% of patients in large series [89]. Four randomized controlled trials (RCTs) [90–93] and decades of experience concretize colchicine’s safety and efficacy in FMF treatment. The risk of amyloidosis in patients who adhere to colchicine therapy is less than 1% in the long term – even in the absence of a complete control of attacks [88]. Therefore, lifetime colchicine prophylaxis is recommended in all FMF patients, regardless of symptoms, unless a severe side effect ensues.

Colchicine is an ancient agent used for centuries for the treatment of gout. Although its precise mechanism of action is unknown, colchicine has inhibitory functions on microtubule polymerization, adhesion molecules, neutrophil chemotaxis, and NLRP3 inflammasome [94]. It is thought to exert these effects primarily on neutrophils due to selected accumulation in these cells as they lack the P glycoprotein efflux pump [95]. Colchicine’s bio-availability ranges from 24% to 88% while its elimination half-life ranges from 20 to 40 h and is prolonged in patients with liver and kidney impairment [94]. It has a narrow therapeutic index while being clinically effective in most FMF patients at blood concentrations <7 ng/mL, but it can cause serious toxicities, multiple organ failure, and death at concentrations >10 ng/mL [96]. Oral doses of 1–1.5 mg/day is the optimal dose in most patients for the prevention of attacks and AA amyloidosis [22,97]. But the dosage can be increased to a maximum of 2.5–3 mg/day if the response is inadequate [97–99]. In previous long-term studies with large number of pediatric and adult patients, titrating the dose to control symptoms resulted in less than 1% risk of proteinuria [98]. However, it’s prudent to keep dosage ≥1.5 mg/day in patients with or at high risk of AA amyloidosis, provided that the liver and kidney functions are normal [100]. The dose can be taken once or divided according to gastrointestinal tolerance. Colchicine is safe and even improves pregnancy outcome, so it must be continued during pregnancy and while breastfeeding [96, 98]

The foremost side effects of colchicine are diarrhoea, elevation in transaminases, leukopenia, and neuromuscular toxicity – which all require regular monitoring. Colchicine strongly interacts with tubulin, cytochrome P3A4 (CYP3A4), and P-glycoprotein. Therefore, the risk of toxicity increases when colchicine is combined with drugs or foods which are substrates of these proteins. Many fatalities have been reported with the combined use of clarithromycin, cyclosporin, lipid-lowering drugs, and grapefruit [94]. Howbeit, colchicine is generally safe, requiring permanent cessation in 2% of patients in long term [101]. Nonetheless, colchicine intolerance constitutes a major problem, with almost one-fifth of patients unable to maintain optimal doses [101]. In a large cohort, diarrhoea (10.8%), transaminitis (5.9%), leukopenia (1.1%), renal impairment (1.3%), myopathy (0.5%), and occasional skin side effects hindered optimal colchicine dosing [101]. Of note colchicine frequently unmasks lactose intolerance and GI side effects can often be ameliorated by introduction of a lactose free diet, particularly in those with a high milk or other dairy product intake.

Ultimately, colchicine at doses of 1–2 mg/day, titrated according to response, has provided 64% complete and 31% partial response in the long term [102]. However, about 5%–10% of patients do not respond well to colchicine, requiring additional treatment [102]. Patients with M694V homozygous or biallelic exon 10 variants, severe disease, or musculoskeletal and skin manifestations are more likely to be colchicine-nonresponsive, whereas patients with serositis or fever-type attacks and single-heterozygous variants may respond to lower colchicine doses [103]. Colchicine cessation can be possible in the latter group provided that they are in complete remission for at least for 3–5 years, has normal acute phase levels, at low risk of AA amyloidosis and adhere with regular follow up [104,105]. However, most physicians prefer lifelong colchicine prophylaxis. Nevertheless, colchicine-free long-term remission is possible in minority of patients [104].

### 9.1. Treatment of colchicine-resistant or -intolerant patients

No clear consensus offers a precise definition of ‘colchicine resistance’, but experiencing monthly attacks or persistently elevated inflammation markers despite the adherence of a maximally tolerated colchicine is the most widely accepted definition [99,106]. Colchicine-resistant (crFMF) or colchicine-intolerant (ciFMF) patients are candidates for biologic therapies, but guidelines for their use are lacking. Therefore, before starting biologics, there are several issues to consider: patients’ quality of life, work productivity and disposition, the presence of chronic inflammation, and reimbursement for drug costs. Furthermore, causes of nonresponse should be carefully reviewed, including attack mimics, comorbid conditions, potent attack inducers, malabsorption and, most importantly, treatment adherence [96,107]. Avoidance of precipitating factors – such as cold exposure, strenuous exercise, certain drugs, and certain foods – might reduce the frequency of attacks. A prospective observation period of three to six months to confirm attacks and determine inciting factors is recommended before the use of biologic drugs [108]. 

Three IL-1 antagonists have been studied in the treatment of crFMF: anakinra, canakinumab, and rilonacept. All of the IL-1 antagonists significantly reduce the number of attacks and improve quality of life with good safety profiles [108,109]. Good quality studies are scarce for the use of IL-1 antagonists and all randomized or open label studies included relatively small numbers of patients. Most of the evidence comes from single case reports or case series. The most commonly used drugs for crFMF are anakinra and canakinumab [110]. There are no comparative studies between agents for canakinumab vs. anakinra, so both agents can be used for the management of crFMF and ciFMF patients. Their long-term efficacy and safety are unknown, but they are effective in stabilizing and improving FMF complications such as proteinuria in the short term, suggesting potential benefits in the long term [111,112]. Hence, until the long-term effects of IL-1 antagonists are demonstrated, colchicine should be coadministered unless it is contra-indicated. The rate of infection is low with use of IL-1 antagonists and the main limitation for their use is their financial expense. 

**Figure 6:**
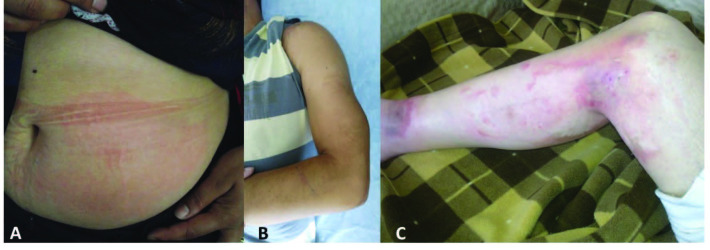
Side effects of anakinra (A) injection site reaction, (B) lipoatrophy, (C) toxic epidermal necrolysis.

Anakinra, a recombinant IL-1 receptor antagonist (rHIL-1Ra), was the first IL-1 blocking biologic agent produced and inhibits binding of both IL-1α and IL-1β to the IL-1 receptor. It is the most extensively used IL-1 blocker in FMF treatment and, currently, the cheapest one [108]. Anakinra was studied in only one RCT included 25 FMF patients (12 in anakinra and 13 in placebo arms). All 12 patients responded, and seven (58.3%) were complete responders [113]. Anakinra has a relatively short half-life (4–6 h) requiring daily injections. An advantage of the short half-life is that it can be used as a diagnostic challenge test for the suspected IL-1 mediated diseases [114] and also to determine whether an unusual manifestation relates to FMF itself [115]. Moreover, anakinra’s short half-life allows its use in the preattack prodromal period to alleviate the symptoms of an impending attack. The prophylactic, intermittent, short-period use of anakinra before exposure to known predictable triggers, such as menstruation and travel, might also be effective [116]. The recommended dose for anakinra is 1–2 mg/kg/day with a maximum daily dose of 8 mg/kg. The usual treatment dose is 100 mg/day, and the dose can be escalated according to patient response – though multiple doses are hardly tolerated by patients due to painful injections. The mean plasma clearance of anakinra decreases by 70%–75% in patients with end-stage renal disease (ESRD), requiring a dosage adjustment (i.e. starting with a dose of 100 mg every other day). Other significant side effects are skin reactions, leukopenia, infections, and weight gain (Figure 6). Loss of efficacy may occur in long-term use, which is an indication for switching to canakinumab treatment [110]. 

Canakinumab differs from anakinra in that it specifically blocks IL-1β. It has a mean plasma half-life of 26 days, allowing for monthly or bimonthly administrations. Canakinumab is the only licensed drug in Europe and the United States for the treatment of crFMF. Two open-label trials and one RCT evaluated canakinumab’s efficacy. Once-every-four-weeks doses of 150 mg (2 mg/kg in children) of canakinumab provided a complete response of 61% in CLUSTER trial, and when the dose doubled for patients with partial responses (300 mg in adults, 4 mg/kg in children, every four weeks), the complete response rate increased to 71% [117]. In light of this study, canakinumab is started at 150 mg/month and, according to response, the dose can increase to a maximum of 300 mg/month or taper to 150 mg every other month. In daily practice, canakinumab injections are more comfortable than anakinra injections, which might improve treatment compliance [110]. 

Rilonacept, an IL-1 decoy receptor, binds to IL-1β and – to a lesser extent – IL-1α. Rilonacept is the least-studied and least-used IL-1 antagonist in FMF treatment. In a small RCT comprising 14 patients, two patients had a complete response while eight had a partial response and two had no response during a three-month treatment course [118].

Tocilizumab is a humanized monoclonal antibody against the interleukin-6 receptor, and it has potent suppressive effects in the production of APRs. Although FMF is considered an IL-1 mediated disease, serum IL-6 concentrations have been found to be elevated in FMF patients, particularly during attacks [119]. Yilmaz et al. treated 11 FMF amyloidosis patients with a monthly infusion of 8 mg/kg of tocilizumab for a duration of three to 16 months. Proteinuria improved or stabilized in eight patients, and no attacks were observed in ten patients [120]. After proteinuria normalization, the researchers discontinued tocilizumab in two patients, which resulted in a relapse of proteinuria for both patients [121]. Another study by Ugurlu et al. observed similar results [122]. Ongoing RCTs in Germany (NCT03446209) and Japan (UMIN000028010) will explore role of tocilizumab in treatment of crFMF and ciFMF patients. 

### 9.2. Treatment of acute episodes

The therapeutic approach to an acute FMF episode is mainly supportive, including the administration of intravenous saline for hydration and the use of analgesics [97]. However, it has been suggested that the use of anakinra in the prodromal period might abrogate or alleviate the symptoms of an imminent attack [31,116]. Many patients report antecedent precipitating insults before attacks, and 70% of these insults were followed by an attack [123]. Therefore, use of anakinra soon after a powerful attack trigger would also be a reasonable approach. Anakinra can be used in acute attacks which are potentially life threatening, expected to be last longer or leave permanent damage, such as pericarditis, febrile myalgia and hip joint arthritis. 

### 9.3. Treatment of comorbidities

As we mentioned earlier, various inflammatory conditions may accompany FMF. The treatment of these conditions is the same when they accompany FMF as their usual treatment in non-FMF patients. However, some of the successful reported treatments are disclosed in Table 3. Interestingly, some of the inflammatory comorbid conditions in the course of FMF may also respond well to IL-1 inhibitors, such as spondyloarthritis, Behçet’s disease, inflammatory bowel disease, protracted febrile myalgia, hidradenitis suppurativa, pustular psoriasis, and HSP [108]. 

**Table 3:** Selected comorbidities and reported successful treatments.

Condition	Treatment(s)	Study	Reference
Chronic arthritis	DMARDs	Case series	[143,144]
	Anti-IL-1	Case series	[110]
	Anti-TNF	Case series	[145,146]
	Tofacitinib	Case series	[147]
Sacroiliitis	Anti-TNF	Case series	[146]
	Anti-IL-1	Case series	[148]
AA Amyloidosis	Anti-IL-1	Case series	[111,112,149,150]
	Anti-IL-6	Case series	[120,122, 151–154]
	Interferon-α2a	Case	[155]
	Anti-TNF	Case series	[156,157]
PFM	Anti-IL-1	Case series	[46,158,159]

DMARDs: Disease modifying antirheumatic drugs, IL: Interleukin, PFM: Protracted febrile myalgia, TNF: Tumor necrosis factor.

Chronic arthritis in FMF does not respond to colchicine alone and requires additional medications, such as disease-modifying antirheumatic drugs (DMARDs), intraarticular steroid injections, or biologics (Table 3). Similarly, protracted febrile myalgia is resistant to colchicine, which can be treated with glucocorticoids, NSAIDs, and IL-1 antagonists. 

### 9.4. Treatment of complications

Colchicine can stabilize or even improve proteinuria in FMF-associated amyloidosis in colchicine-naïve or noncompliant patients with serum creatinine levels of <1.5 mg/dL, and the dose should not be less than 1.5 mg/day for such patients [100] though dose adjustment is necessary in the case of reduced creatinine clearance. Intensive blood pressure control should be maintained in all proteinuric subjects along with anti-lipidemic treatments. There is a significant correlation between serum SAA concentration and increased risk of death from amyloidosis, with ~4-, ~5-, and ~12-folds for values above 4, ~10, and ~45 mg/L, respectively [84]. Therefore, for patients with AA amyloidosis and ongoing inflammatory activity (preferably assessed with SAA or CRP) despite the maximum tolerated dose of colchicine, biologic treatments should be promptly initiated. The target SAA value would be less than 10 mg/L, since this cut-off has a 60% chance of regression in amyloid deposits and improved survival [84]. If SAA is not available, CRP values 5 mg/L in FMF children or 8.75 mg/L in FMF adults during attack-free periods can be a convenient substitute to guide therapeutic decisions [124]. The use of IL-1 inhibitors improves AA amyloidosis patients’ renal function [111,112]. 

End-stage renal disease caused by renal amyloidosis should be treated the same way as other causes of renal failure, including transplantation. After kidney transplantation, tight control of inflammation should be maintained, and colchicine drug interactions must be carefully assessed. Tight inflammation control may also improve complications other than AA amyloidosis, such as growth retardation and infertility. The use of IL-1 inhibitors has been shown to restore fertility in FMF patients suffering from infertility or subfertility [125].

### 9.5. Approach to patients with chronic subclinical inflammation

Chronic inflammation constitutes a substantial risk for AA amyloidosis, growth retardation, and cardiovascular disease [54]. In such cases, FMF disease activity must be carefully assessed using prospective patient diaries and in confirmed quiescent patients, all other possible causes of chronic inflammation should be ruled out, including inflammatory comorbidities and chronic infections [55]. In true asymptomatic patients, colchicine dose must be increased/maintained at maximum tolerated doses until SAA normalizes which has been demonstrated previously [98, 126]. Currently, there is no evidence to support routinely recommending IL-1 or IL-6 antagonists to patients in clinical remission but with elevated APRs. However, a vigilant follow-up for proteinuria and promptly initiating IL-1 or IL-6 antagonists when microalbuminuria is detected, and use of them in those at high risk of AA amyloidosis (presence of several risk factors) would be the reasonable approaches. One issue is that even in patients at high risk of amyloidosis, acceptance and adherence to IL-1 antagonists in the absence of symptoms is low. 

## 10. Prognosis

FMF has an excellent prognosis if diagnosed and treated early, before complications develop. Serious complications almost always develop in colchicine-uncompliant or -resistant cases or among patients with delayed diagnoses. In a recent study, we found in a large cohort that more than half of FMF patients suffer from at least one disease complication [127], assessed by autoinflammatory disease damage index (ADDI) [5]. Damage mainly occur in the musculoskeletal, reproductive, and excretory systems – including growth retardation, pubertal delay, amenorrhea, infertility or subfertility, proteinuria, AA amyloidosis, kidney failure, serosal scarring and adhesions, musculoskeletal pain, joint deformity, and osteoporosis[127]. Risk factors for damage are M694V homozygosity, colchicine resistance/nonadherence, musculoskeletal attacks, diagnostic delay, disease duration and chronic inflammation [127]. Systemic AA amyloidosis is the leading cause of morbidity and mortality in FMF [128,129], therefore patients with defined risk factors should be meticulously followed (Table 4). After the diagnosis of AA amyloidosis, end stage renal disease develops in median five years and the 5-year survival rate was reported as 50% [130,131]. Allograft survival after transplantation is poorer in FMF associated amyloidosis than other causes of ESRD [132, 133]. 

**Table 4:** Risk factors for AA amyloidosis.

Characteristics	Fold risk increase	Reference
Male sex	1.7– 4	[67,160]
Arthritis	2.3 – 2.4	[127,160,161]
Very frequent attacks ≥20 per year	2.0	[74]
Family history of AA amyloidosis	2.0	[74]
Chronic inflammation	3.59–13	[54,55]
M694V homozygosity	2.6–4.3	[74,160]
SAA1 α/α genotype	3–6.9	[67,160]
Living in Turkey, Middle East, Armenia vs. Europe	1.5–3.2	[74]

Poor sleep quality, anxiety, depression and fatigue are frequently neglected health issues in FMF patients [134,135]. Work productivity is impaired in severe disease and can be improved with good control of disease with colchicine and IL-1 antagonists. Reports have suggested that FMF patients have significantly lower incidence of cancer than the general population [136, 137], raising speculations that MEFV can act as an oncosuppressor gene [138]. On the other hand, numerous reported cases describe mesothelioma in FMF, attributed to chronic serosal inflammation [138]. 

## 11. Follow-up

After an FMF diagnosis, all of the patient’s family members should be evaluated for the disease. Colchicine should be initiated immediately upon a clinical FMF diagnosis. Response, toxicity, and compliance should be monitored every three to six months. Persistence of attacks or subclinical inflammation represents disease activity and an indication to increase colchicine dosage [99]. Monitoring disease activity with a diary is probably the best way to assess the frequency and duration of attacks and to ascertain precipitating factors. Paper and mobile phone application versions (AIDD) of patient diaries exist, but patient adherence is poor. Wearable technology could be a promising tool to monitor disease activity by capturing attacks based on abrupt decline in routine physical activity [139]. 

Complete blood count, liver enzymes, creatinine, CRP or SAA, and urinalysis should be monitored every three to six months to monitor colchicine toxicity, inflammatory activity, and proteinuria [99]. A kidney biopsy is recommended whenever the urinary protein excretion exceeds 0.5 g/24 h to exclude AA amyloidosis [140]. More convenient sites for the tissue biopsies are abdominal fat and minor salivary glands [141]. The other option for patients with suspected amyloidosis is the use of a serum amyloid P (SAP) scan but it is only available at certain centers [142]. 

## 12. Future perspectives

Genetic testing, combined with functional assays, might be the diagnostic strategy in the near future, and it may also aid in treatment planning. Extension studies will disclose the long-term benefits of IL-1 antagonists in terms of preventing damage. Clinical trials could, moreover, investigate the efficacy of caspase inhibitors and more potent colchicine derivatives. Since FMF commonly results from missense mutations, CRISPR/Cas9 system could potentially provide a cure in the future. 

## 13. Conclusion

FMF is an enigmatic disease in both its pathogenesis and clinical manifestations. Although FMF manifests with intermittent attacks, many patients suffer from complications of disease. Early diagnosis and adherence to treatment are key issues for the well-being of patients. Although the last 25 years has seen major progress regarding the pathogenesis, diagnosis and treatment of FMF, there are many questions awaiting their answers such as episodic nature of disease, variants of unknown significance, epigenetics and rational use of biologic drugs. 

## Disclaimers

Abdurrahman Tufan has received research grants from Pfizer Inc., and Novartis Pharmaceuticals.
